# Dry Matter Production, Nutrient Cycled and Removed, and Soil Fertility Changes in Yam-Based Cropping Systems with Herbaceous Legumes in the Guinea-Sudan Zone of Benin

**DOI:** 10.1155/2016/5212563

**Published:** 2016-06-30

**Authors:** Raphiou Maliki, Brice Sinsin, Anne Floquet, Denis Cornet, Eric Malezieux, Philippe Vernier

**Affiliations:** ^1^Institut National des Recherches Agricoles du Bénin (INRAB), P.O. Box 2128, Calavi, Benin; ^2^Faculté des Sciences Agronomiques de l'Université d'Abomey-Calavi (FSA/UAC), P.O. Box 01-526, Cotonou, Benin; ^3^Centre Béninois pour l'Environnement et le Développement Economique et Social (CEBEDES), P.O. Box 02-331, Cotonou, Benin; ^4^Centre de Coopération Internationale en Recherche Agronomique pour le Développement (CIRAD), 34398 Montpellier Cedex 5, France; ^5^Centre de Coopération Internationale en Recherche Agronomique pour le Développement (CIRAD), UPR Hortsys, 34398 Montpellier Cedex 5, France

## Abstract

Traditional yam-based cropping systems (shifting cultivation, slash-and-burn, and short fallow) often result in deforestation and soil nutrient depletion. The objective of this study was to determine the impact of yam-based systems with herbaceous legumes on dry matter (DM) production (tubers, shoots), nutrients removed and recycled, and the soil fertility changes. We compared smallholders' traditional systems (1-year fallow of* Andropogon gayanus*-yam rotation, maize-yam rotation) with yam-based systems integrated herbaceous legumes (*Aeschynomene histrix*/maize intercropping-yam rotation,* Mucuna pruriens*/maize intercropping-yam rotation). The experiment was conducted during the 2002 and 2004 cropping seasons with 32 farmers, eight in each site. For each of them, a randomized complete block design with four treatments and four replicates was carried out using a partial nested model with five factors: Year, Replicate, Farmer, Site, and Treatment. Analysis of variance (ANOVA) using the general linear model (GLM) procedure was applied to the dry matter (DM) production (tubers, shoots), nutrient contribution to the systems, and soil properties at depths 0–10 and 10–20 cm. DM removed and recycled, total N, P, and K recycled or removed, and soil chemical properties (SOM, N, P, K, and pH water) were significantly improved on yam-based systems with legumes in comparison with traditional systems.

## 1. Introduction

One of the most serious problems of farming system is the excessive reductions of agricultural productivity resulting from major degradation of soil fertility. In 1990 Edouard Saouma wrote that the most serious problem of African countries in the future can be that of land degradation [1]. To understand how and why lands become degraded, one needs some knowledge of the physical environment, population, cultivation history, and farming systems [2, 3].

Current yam-based cropping systems, which involve shifting cultivation, slash-and-burn, or short fallow, often result in deforestation and soil nutrient depletion [4]. As long as population pressure was low, the cropping phase was short compared to the fallow period. Three or four years of cultivation followed by ten years or more of fallow, for example, allows the accumulation of easily degradable organic matter to regenerate soil fertility [5, 6]. Where population increases, available land per inhabitant is reduced and fallow periods shorten. Traditional long-fallow shifting cultivation can no longer continue in most of humid Sub-Saharan Africa. Increasing population densities are posing a serious threat to natural resources and agricultural production. Farmers' response to higher food demand has been either an increase in cultivated area or a reduction of fallow period. The minimum fallow duration to maintain crop production was estimated at 12 years [7]. Fallow periods in most of the humid zone of West and Central Africa are actually between 2 and 5 years [8], reinforcing the need to seek alternative food production systems [7].

Yam (*Dioscorea* spp.) is a tuber crop widely cultivated in the humid and subhumid lowland regions of West Africa and the Caribbean [9]. More than 90% of the worldwide production (40 Mt fresh tubers/year) is produced in West Africa [10]. Yam is grown in traditional cropping systems as the first crop after land clearance, yielding about 10 t of fresh tuber/ha/year [11], but when the soil fertility is high, it can easily reach 25–30 t/ha in farm fields [12] with* Dioscorea cayenensis-rotundata* varieties. In Benin nowadays, farmers hardly have the possibility to rely on long duration fallow and yam is being cultivated in 1- or 2-year herbaceous fallow or maize rotation systems with manual incorporation of residue into the soil [13, 14]. Smallholder farmers removed important quantities of nutrient from their soil without applying a sufficient quantity of manure or fertilizer to replenish the soil [15].

Yam cultivation in West Africa is now confronted with the scarcity of fertile soil available for clearing [4]. In Benin nowadays, farmers hardly have the possibility to rely on long duration fallow and yam is being cultivated in 1- or 2-year herbaceous fallow-yam or maize-yam rotation systems with manual incorporation of residue into the soil [13, 14]. Smallholder farmers removed important quantities of nutrient from their soil without applying a sufficient quantity of manure or fertilizer to replenish the soil [15].

The decline in yam yields under continuous cultivation has led to the largely accepted conclusion that yam requires a high level of natural soil fertility (organic matter and nutrient) [16]. Since the demand for yam keeps increasing due to the continued population growth, reserves of arable land are diminishing, and fallow duration is decreasing. It is becoming necessary to sustainably increase yam productivity in sedentary cropping systems [16]. There is a dire need therefore to assess in farmers' conditions the economic performance of sustainable cultivation techniques. Ongoing soil degradation could be reduced by the adoption of new farming techniques such as improved fallows of herbaceous legumes [17, 18].

Studies on improved fallow practices are generally grain-oriented (cereals, such as maize), whereas very little has been done on root and tuber crops, especially yam. Comparative studies are lacking that assess the effects of yam-based technologies with herbaceous legumes intercrops and short fallows on yam production and soil properties in the savannah transition agroecological zone of Benin. We compared in a perennial experiment for 4 years, with 2-year rotations, smallholder farmers' traditional rotations maize-yam or 1-year* Andropogon gayanus* fallow-yam with rotations intercropped* Aeschynomene histrix* with maize-yam or intercropped* Mucuna pruriens* with maize-yam. The objective of this study was to determine the impact of yam-based systems with herbaceous legumes on dry matter (DM) production (tubers and shoots), nutrients removed and recycled, and the soil fertility changes.

## 2. Materials and Methods

### 2.1. Study Sites

**Figure 1:**
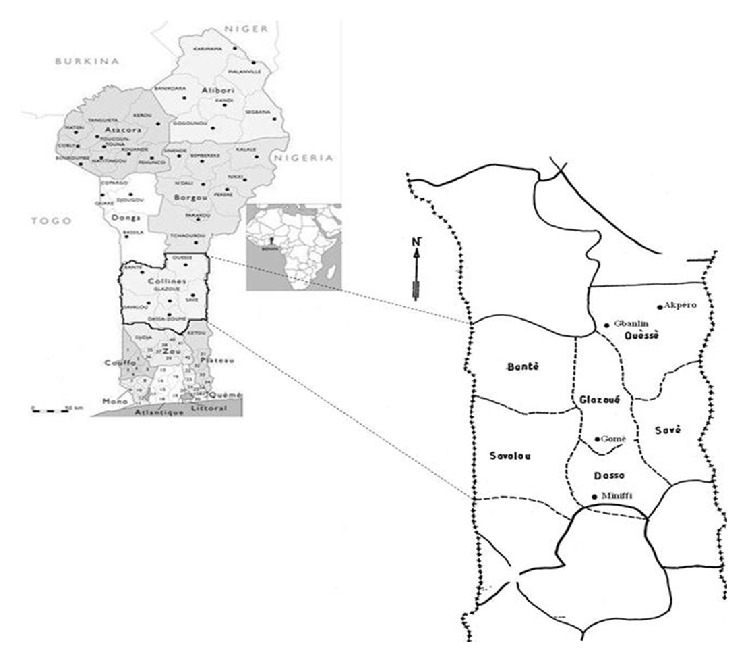
Study area location in the savannah transitional agroecological zone of Benin.

The study was carried out in the Guinea-Sudan transition zone of Benin (centre of Benin) in four sites: Miniffi (District of Dassa-Zoumè), Gomè (Glazoué), Akpéro, and Gbanlin (Ouessè) with latitudes 7°45′ and 8°40′ north and longitudes 2°20′ and 2°35′ east (Figure 1).

The climate is tropical transitional Guinea-Sudan with a rainfall distribution gradient from bimodal (Southern Benin) to monomodal (Northern Benin). The average annual rainfall during the study period was 1052 mm (2002), 1386 mm (2003), 983 mm (2004), and 797 mm (2005). The rainfall regime in the study area is variable and unequal distribution (i.e., number of rainy days per month) varies from one site to another. The 2002 and 2003 cropping seasons were wet and had better rainfall distribution with an average annual precipitation of 1200 mm, whereas 2004 and 2005 were dry (890 mm) with relatively low rainfall distribution.

Most of the soils are tropical ferruginous soils [19], originally from Precambrian crystalline rocks (granite and gneiss), and classified as plinthosols (Gbanlin and Akpéro) and luvisols (Miniffi and Gomè) [20] (Table 1). Miniffi, Akpéro, and Gbanlin are located on a plateau while Gomè is on lowland. Akpéro is close to forest while Gbanlin, Miniffi, and Gomè are far. There is a rising gradient of fertility from the continuous cropping system on degraded soils towards the forests. This degradation is related to soil organic matter decrease, which leads to nutrient depletion (nutrients removed in the crop harvest, leaching, and erosion). Vegetation is a degraded woody savannah type. Maize, yam, cassava, and groundnut are annual cropping systems and the cash crops are cotton and soybean. Mineral fertilizer application appears to be essential. Smallholder farmers use fertilizers on maize on depleted soils depending on cash and inputs availability. Cotton is not mixed cropping, but pure crop in rotation with other crops (maize or sorghum).

### 2.2. On-Farm Experiment

The concept of the experiment was to produce residue biomass followed by planting yam in rotation cropping systems. A previous cover crop (fallows or intercropped maize/legume) was designed to provide organic matter for the following main crop (yam) (Table 1).

Smallholders carried out two-year rotations experiment of yam-based cropping systems repeated twice (2002–2005) on-farm with single-harvest late maturing variety of yam “Kokoro” (*Dioscorea rotundata*). This is one of the most cultivated species in the study area due to its good aptitude for conservation and processing into dried tubers (the so-called chips), flour, and starchy paste (locally called* amala*) [21]. We conducted the experiment with 32 farmers, eight in each site (Miniffi, Gomè, Akpéro, and Gbanlin). For each of them, we used a randomized block design with four replications and four levels of treatment. Plot size was 10 m × 10 m (total of 1 600 m^2^ per farm). The four treatments were as follows:

- *T0 (Control 1)*. T0 is one-year fallow-yam rotation, which is a common practice in the area. A natural fallow of* Andropogon gayanus* grass was grown in the first year.
- *TM (Control 2)*. TM indicates maize-yam rotation, which is also a common practice in the area. Maize was planted (spacing 80 cm × 40 cm) in April of the first year.
- With recurring drought stress exacerbated by highly variable and unpredictable rains in the study area, some farmers grow a second crop, which often fails. This corroborates the great interest of the maize/leguminous crop when no second crop is planned.
- *TMA*. TMA is intercropped* Aeschynomene histrix* with maize-yam rotation: maize was planted in April of the first year.* A. histrix* seeds (7 kg ha^−1^) were mixed with dry sand (3/4 sand and 1/4 seeds) and sown two weeks after the maize.
- *TMM*. TMM is intercropped* Mucuna pruriens* with maize-yam rotation: maize was planted in April of the first year.* M. pruriens* seeds (25 kg ha^−1^) were sown at spacing 80 cm × 40 cm in May six weeks after the maize.

On treatments TM, TMA, and TMM, 100 kg ha^−1^ NPK fertilizer (14% N, 10% P, and 11.7% K) was applied to maize in April and 50 kg ha^−1^ urea (46% N) in June. The maize was harvested in July. The grainless* M. pruriens* and* A. histrix* crops were mowed 140 and 180 days, respectively, after planting. Organic matter was incorporated in moulds and left on the surface as mulch in October and then yam was planted directly on these moulds, without mineral fertilization.

### 2.3. Data Collection

Composite soil samples were collected in each field before the beginning of the experiment along plot transects at soil depths of 0–10 cm and 10–20 cm (32 farm fields × 2 depths = 64 samples) in order to determine soil characteristics. At the end of 2005 before yam harvesting, composite soil samples were collected at the same depths in the moulds along plot transects (32 farm fields × 4 treatments × 2 depths = 256 samples).

Prior to ridging, in four 1 m^2^ quadrats within each plot the aboveground biomass of herbaceous legumes and fallow was collected in October 2002 and 2004. The biomass samples were dried at 60°C until constant weight and then dry weight was determined. At maturity, maize grain and stover were harvested per row on each plot and dry matter (DM) determined. DM of yam tubers and shoots was estimated on each plot in December 2003 and 2005 (Tables 2 and 3).

### 2.4. Soil and Plant Nutrients Content

The nutrients contents of the soil samples were performed in the Laboratory of Soil Sciences, Water and Environment (LSSEE) of INRAB (Benin National Research Institute). The plant nutrient content was estimated according to the biomass amount.

Soil and plant macronutrients content (N, P, and K) were analyzed. Nitrogen (N) content was analyzed using the Kjeldahl method [22], available phosphorus with Bray 1 method [23], potassium with the FAO method [24, 25], organic carbon with the Walkley and Black method [26], and soil fractionation with Robinson method [27] and pH (H_2_O) (using a glass electrode in 1 : 2.5 v/v soil solution). Only yam tuber and maize grain were removed, and all other plants parts were recycled (*A. gayanus*, maize stover, yam shoot,* A. histrix,* and* M. pruriens*). Yam or* M. pruriens* shoot included leaves. Nutrient removed or recycled was calculated as a summation of nutrient concentration time dry matter of the respective plant parts. Dry matter removed or recycled was calculated as a summation of dry matter of the respective plant parts.

### 2.5. Analyses of Variance to Test the Effect of Site, Year, and Treatment on Yam Yield

Analysis of variance (ANOVA) using the general linear model (GLM) procedure [28] was applied to the DM production (tubers and shoots), nutrient contribution to the systems, and soil properties at depths 0–10 and 10–20 cm. The experiment was conducted with 32 farmers, eight in each site. For each of them, a randomized complete block design with four treatments and four replicates was carried out using a partial nested model with five factors: Year, Replicate, Farmer, Site, and Treatment. The random factors were “Year” and “Replicate” and “Farmer.” Farmer was considered as nested within “Site” and “Replicate” as nested within “Farmer.” The fixed factors were “Treatment” and “Site.” Sites were considered as fixed based on certain criteria such as landscape (lowland and plateau), soil type, and initial soil fertility. Yield values were logarithmically transformed to normalize the data and to stabilize population variance. The GLM was computed to assess the interactions between the factors involved. Least square means and standard error were also computed for factor levels, and the Newman and Keuls test was applied for differences between treatments. Significance was regarded at *P* ≤ 0.05.

## 3. Results

### 3.1. Initial Soil Characteristics

The relevant general soil physical and chemical characteristics before are presented in Table 4.

Site physical characteristics such as soil texture (sand) were relatively high (74.778%–88.79%) followed by silt (5.55%–17.36%) and clay (5.66%–7.861%) with the lowest content. The soils had a neutral reaction, with pH (H2O) ranging from 6.3 to 6.8.

The initial soil fertility status of different sites was low. Soil organic matter (SOM) contents were low in all fields, ranging from 0.93% to 2.258%, and the C : N ratio ranged from 8.69 to 11.70. Available P levels were very low and varied from 3.012 to 20.125 mg/kg-soil. Soil N concentration ranged from 0.056% to 0.112%. N, P, and SOM contents were significantly higher in 0–10 cm than in 10–20 cm depth, except at Gbanlin site for N and SOM. Gomè site showed, for both soil depths, the lowest values of carbon (C%), N%, P (mg/kg-soil), and organic matter (%), whereas Akpéro had the highest values.

### 3.2. Dry Matter Production and Nutrient Contribution to the Systems

In the 2002 and 2004 cropping seasons, the highest biomass dry matter (DM) amount recycled was recorded on TMM (Table 5).

The ANOVA partial nested model shows that yam yield DM differed significantly depending on the factor Treatment (*P* < 0.001). The factors Site and Year were not significant for yam yields DM. But Replicate (*P* < 0.001), Treatment × Farmer (*P* < 0.01), and Year × Farmer interactions (*P* < 0.001) were significant (Table 6).

Dry matter (t ha^−1^) of yam tubers removed and yam shoots recycled, N, P, and K content (kg ha^−1^) dry matter of plant parts removed in the crop harvest, and those returned to the soil in yam-based cropping systems were significantly higher in TMA and TMM than in T0 and TM during both cropping seasons (Tables 7 and 8).

Therefore, total plant N, P, and K (kg ha^−1^) dry matter removed in the crop harvest and those returned to the soil in yam-based cropping systems were significantly higher in TMA and TMM than in T0 and TM during both cropping seasons (Table 9).

### 3.3. Effects of Treatments on Soil Characteristics

Afterwards soil characteristics at the end of the experiment globally showed relatively low clay, silt, and relatively high sand concentration on different sites under different treatments (T0, TM, TMA, and TMM) in comparison with initial soil characteristics at the beginning of the experiment. Soil organic matter concentration was improved at 10–20 cm depth particularly in Miniffi (1.247%, 1.176%, 1.326%, and 1.409%) on T0, TM, TMA, and TMM, respectively, and Gomè (1.010%, 0.959%, 1.046%, and 1.126%). Globally, soil N and P concentrations were improved on different sites on treatments TMA and TMM in 0–10 cm or 10–20 cm depth (Tables 10(a)–10(d)).

The end of study soil analysis showed soil chemical properties (SOM%, N%, P (mg/kg-soil), K^+^ cmol kg^−1^, and pH water) significantly higher in TMA and TMM than in traditional systems T0 and TM (*P* < 0.001). Soil clay contents were significantly higher in TMA, TMM, and T0 than in TM (*P* < 0.001). No significant difference was observed for silt and sand concentrations for different treatments (Table 10(e)).

## 4. Discussion

### 4.1. Dry Matter and Nutrients Recycled in Yam-Based Cropping Systems

The highest biomass dry matter (DM) amount recycled was recorded on* Mucuna/*maize intercropping (TMM).* Mucuna* grows rapidly and DM production can reach 10 t ha^−1^ [2, 17, 29]. In fact,* Mucuna* creeps and climbs maize straw in pattern crop allowing the lianas staking. Therefore,* Mucuna* large leaves profit from solar radiations improving the photosynthetic activity and the plant productivity.* Mucuna* reaches the physiological maturity (flowering time) between 180 and 240 days after grains planting in the study area in comparison with* Aeschynomene* (200–306 days) [30, 31].

DM of yam shoots recycled on TMA and TMM were significantly higher in 2005 (dry year) than in 2003 (humid year). The chemical fertilizers applied and the above biomass DM of intercropping maize and herbaceous legume recycled and accumulated in 2002, 2003, and 2004 could have resulted in a combined beneficial effect of water, nutrient use, and plant growth in 2005. DM amounts of* M. pruriens*,* A. histrix,* and maize stover recycled were higher in 2002-2003 (humid year) than in 2004-2005 (dry year). In fact, plant yields and agronomic productivity were constrained by recurring drought stress exacerbated by highly variable and unpredictable rains.* M. pruriens* stover showed the highest DM amount followed by* A. histrix* whatever the year and this could reach 10 t ha^−1^ [18] because* M. Pruriens,* compared with* A. histrix,* grows more rapidly and close.

The nutrient (N, P, and K) levels removed or recycled fit the DM production (tubers and shoots) and then varied according to treatment and cropping season.

### 4.2. Impact of Yam-Based Cropping Systems with Herbaceous Legumes on Soil Properties

Most of the soils as mentioned above are tropical ferruginous soils, originally from Precambrian crystalline rocks (granite and gneiss) and classified as plinthosols (Gbanlin and Akpéro) and luvisols (Miniffi and Gomè). Miniffi and Akpéro are located on a plateau (well-drained soils) while Gomè is on lowland (more poorly drained soils). Gbanlin is located on an undulating plateau with concretions. Soil chemical analysis showed that the soil was deficient in N, P, and K and soil organic matter (SOM). This could be due to the mining agriculture and also a consequence of the mechanical destruction of the soil structure during the ridging for yam crop. In fact yam is a demanding crop in terms of organic matter and nutrients. Research [32] reported that yam yielding about 30 t of fresh tuber ha^−1^ removes 120 N kg ha^−1^, 5.1 P kg ha^−1^, and 111 K kg t^−1^. When land is used too intensively, the SOM is rapidly reduced in the unstable fraction. In the short and medium term, this reduction leads to a decrease in soil biological activity and, then, contributes to soil degradation and depletion [33]. Many studies report that soil organic matter (SOM) decreases in cultivated soils [33]. This decrease is linked to the depth of the cultivated soil layer and is probably exacerbated in yam-based cropping systems.

Nitrogen is the most deficient component of these soils grown with low organic matter content. Total nitrogen deficiency of these soils lies in the fact that nitrogen is the only major nutrient that does not exist in the bedrock. Further, the transfer of atmospheric nitrogen to the soil by biological and chemical process is slow. Losses of nitrogen in these soils are common because of the high volatility and solubility of this nutrient. Nitrogen is generated by the breakdown of inherent organic matter and needs to be supplemented with other sources of organic materials or mineral fertilizer. Many studies focusing on these elements conclude that there is an indisputable need to correct the lack of N and P in the soil in Africa [2, 6].

It is possible to reduce or stop ongoing soil degradation and the decrease in yield with such rotations including improved short fallows or intercropping with herbaceous legumes. The use of legumes improves levels of concentration of the soil parameters. The improvement of the clay concentration at the end of the perennial experiment could be due to the process of the composite soil samples collected on the ridges resulting from the brewing of the soil deep layer relatively rich in clay and the soil horizon surface after ridging. Indeed, ridging allows increasing the volume of the soil deep layer and contributes to the incorporation of organic residues into the soil.

Significant differences in total SOM and nutrients increase with treatments TMA and TMM in comparison with T0 and TM could be due to the faster decomposition of fermentable green manure (herbaceous legumes) with low humification coefficient (5%) added to the moderate decomposition of lignified maize stover on relatively degraded soils [34]. Our observations are in agreement with those of [35] who reported that cropping systems and organic manures have the most influence on the SOM. Rotations with* M. pruriens* and* A. histrix* represented a source of easily available N, P, and K for the yam crop which could be related to their faster decomposition and nutrient release, compared with the slower release of nutrients by poorer quality materials such as maize stover and* A. gayanus* grass. In Ghana, studying the effect of cropping sequences with cassava and legume crops, [36] indicated that only 30% of* M. pruriens* litter remained six weeks after incorporation of the biomass. References [37] and [38] that studied the traditional* M. pruriens*-maize rotation in Honduras estimated that 83% of nitrogen produced by a mulch of* M. pruriens* was available for the following maize crop. They also observed that available P remained practically constant, with 15 to 20 mg/kg-soil in the surface horizon in spite of P exports by maize. Reference [38] concluded that the practice of continued rotation with* M. pruriens* and maize prevented soil N depletion for at least 15 years.

Our results showed that legumes improved soil P. Legumes fallows with* M. pruriens* are known especially for improving the quantity of available P fractions in the soil for subsequent crops [39]. Nevertheless, they depend on the inherent P levels in the soils.* M. pruriens* root exudates could solubilize P increasing its availability. In the study of [40], organic materials have also been found to reduce P sorption capacity of soils and increase crop yields in P limiting soils.

The soil K concentrations were improved in our study (Table 4). Reference [3] showed soil K concentration of 0.82 cmol kg^−1^ in the 0–20 cm soil layer and decreasing significantly with cultivation. The rate of decline was about 0.023–0.054 cmol kg^−1^ year^−1^ in the 0–20 cm soil layer [3].

## 5. Conclusions

The field of interest of the study is to determine the impact of yam-based systems with herbaceous legumes on dry matter production (tubers and shoots), nutrients removed and recycled, and the soil fertility changes. Yam tuber dry matter production was significantly improved in yam-based systems with legumes in comparison with traditional systems. Treatment × Farmer and Year × Treatment interactions influenced significantly the yam tuber dry matter production. Amounts of N, P, and K recycled in yam shoot were significantly higher in yam-based systems with legumes than in traditional systems. The nutrient (N, P, and K) levels removed or recycled fit the DM production (tubers and shoots) and then varied according to treatments and cropping seasons. The end of study soil analysis showed soil chemical properties (SOM%, N%, P (mg/kg-soil), K^+^ cmol/kg, and pH water) significantly higher in treatments with legumes than in traditional systems. We then propose to promote durable and replicable yam-based systems with legumes, through a favorable legislative, economic, and political environment to support local initiatives. Collaborations between farmers, research, development, and extension structures should also be favored to support the development and dissemination of innovations.

## Figures and Tables

**Table 1:** Cropping calendar of yam-based cropping systems with herbaceous legumes and short fallow in the 2002-2003 and 2004-2005 cropping seasons.

	Dec. Jan.	Feb.	March	April	May	June	July	Aug.	Sept.	Oct.	Nov.	Dec.
*2002 or 2004 cropping seasons*												
T0	Natural fallow of *Andropogon gayanus*									Slashing and biomass incorporation (ridging)		

TM	Land slashing and ploughing		Maize planting, NPK application, and weeding			Weeding and urea application	Maize harvesting			Slashing and biomass incorporation (ridging) in furrow		

TMA	Land slashing and ploughing		Maize planting, NPK application, *A. histrix* planting, and weeding			Weeding and urea application	Maize harvesting			Slashing and biomass incorporation (ridging) in furrow		

TMM	Land slashing and ploughing		Maize planting, NPK application, and weeding		*Mucuna* planting	Weeding and urea application	Maize harvesting			Slashing and biomass incorporation (ridging) in furrow		

*2003 or 2005 cropping season*												
T0	Seed yam planting, mounds capping with mulch material yam staking, and weeding					Weeding			Weeding	Yam harvesting		

TM	Seed yam planting, mounds capping with mulch material yam staking, and weeding					Weeding			Weeding	Yam harvesting		

TMA	Seed yam planting, mounds capping with mulch material yam staking, and weeding					Weeding			Weeding	Yam harvesting		

TMM	Seed yam planting, mounds capping with mulch material yam staking, and weeding					Weeding			Weeding	Yam harvesting		

T0: one-year fallow-yam rotation; TM: maize-yam rotation; TMA: *Aeschynomene histrix/*maize intercropping-yam rotation; TMM:* Mucuna pruriens/*maize intercropping-yam rotation.

**Table 2:** Quantity of biomass (t ha^−1^) dry matter and nutrients contents (% and kg ha^−1^) applied in each plot in the 2002 cropping seasons, four village sites (Miniffi, Gomè, Gbanlin, and Akpéro), Benin.

Site/treatment	DM	N	P	K	N	P	K
	t ha^−1^	%	%	%	kg ha^−1^	kg ha^−1^	kg ha^−1^
Akpéro							
T0	4.1	1.7	0.2	0.5	68.4	7.8	21.2
TM	3.5	1.3	0.1	0.5	45.3	5.2	17.4
TMA	9.6	1.3	0.1	0.5	125.9	14.1	47.6
TMM	10.2	1.7	0.2	0.5	177.8	20.2	53.9

Gbanlin							
T0	4.0	1.0	0.2	0.5	42.2	6.0	20.7
TM	3.5	2.3	0.2	0.6	78.5	8.1	22.1
TMA	9.1	1.5	0.1	0.6	132.3	9.3	56.6
TMM	9.5	1.9	0.2	0.6	180.4	14.3	61.1

Miniffi							
T0	4.3	0.9	0.2	0.6	41.1	6.7	27.6
TM	3.7	1.9	0.1	0.6	70.7	4.8	22.1
TMA	9.3	1.2	0.3	0.6	114.8	26.1	59.7
TMM	9.9	2.4	0.1	0.6	239.6	14.9	63.2

Gomè							
T0	4.0	0.9	0.1	0.5	36.4	5.0	19.6
TM	3.5	2.5	0.1	0.6	86.9	2.6	22.2
TMA	9.0	1.2	0.1	0.4	104.9	5.4	34.2
TMM	9.8	1.6	0.1	0.6	160.1	6.7	60.1

**Table 3:** Quantity of biomass (t ha^−1^) dry matter and nutrients contents (% and kg ha^−1^) applied in each plot in the 2004 cropping seasons, four village sites (Miniffi, Gomè, Gbanlin, and Akpéro), Benin.

Site/treatment	DM	N	P	K	N	P	K
	t ha^−1^	%	%	%	kg ha^−1^	kg ha^−1^	kg ha^−1^
Akpéro							
T0	4.3	1.7	0.2	0.5	72.7	8.3	22.4
TM	3.7	1.3	0.1	0.5	47.2	5.4	18.1
TMA	9.3	1.3	0.1	0.5	121.5	13.7	46.0
TMM	10.2	1.7	0.2	0.5	179.7	20.4	54.4

Gbanlin							
T0	4.1	1.0	0.2	0.5	42.7	6.1	21.0
TM	3.5	2.3	0.2	0.6	78.7	8.1	22.2
TMA	9.0	1.5	0.1	0.6	131.0	9.2	56.1
TMM	9.6	1.9	0.2	0.6	182.0	14.4	61.6

Miniffi							
T0	4.0	0.9	0.2	0.6	38.2	6.2	25.5
TM	3.4	1.9	0.1	0.6	65.0	4.4	20.3
TMA	9.4	1.2	0.3	0.6	115.4	26.3	60.1
TMM	10.0	2.4	0.1	0.6	240.2	14.9	63.3

Gomè							
T0	4.0	0.9	0.1	0.5	36.3	5.0	19.5
TM	3.5	2.5	0.1	0.6	86.5	2.6	22.2
TMA	9.3	1.2	0.1	0.4	107.9	5.6	35.2
TMM	9.6	1.6	0.1	0.6	157.3	6.6	59.1

**Table 4:** Initial soil characteristics at the beginning of the experiment at 0–10 and 10–20 cm layers in four village sites (Miniffi, Gomè, Gbanlin, and Akpéro) with 32 farmers, Benin.

	Akpéro		Gbanlin		Miniffi		Gomè	
Depth (cm)	0–10	10–20	0–10	10–20	0–10	10–20	0–10	10–20
	“Plinthosols”		“Plinthosols”		“Luvisols ferriques”		“Luvisols ferriques”	
Clay%	6.58	7.281	5.788	5.66	6.758	6.51	6.828	7.861
Silt%	11.66	11.798	5.808	5.55	6.828	7.081	16.071	17.36
Sand%	81.76	80.920	88.402	88.79	86.412	86.408	77.10	74.778
C%	1.31	1.050	0.69	0.788	0.80	0.64	0.65	0.54
N%	0.112	0.092	0.059	0.081	0.081	0.056	0.073	0.062
C/N	11.70	11.43	11.70	9.68	9.83	11.43	8.90	8.69
OM%	2.25	1.81	1.19	1.36	1.37	1.10	1.12	0.93
PH	6.7	6.7	6.6	6.3	6.7	6.8	6.6	6.6
Bray P	20.125	14.875	7.00	4.00	11.00	3.012	7.987	4.00

C%: soil carbon concentration; N%: soil nitrogen concentration; OM% (=1.72 × C%): soil organic matter content; C/N: index of biodegradability or ratio of soil carbon to nitrogen; Bray P (mg kg^−1^): soil phosphorus.

**Table 5:** Dry matter (t ha^−1^) of plant parts returned to the soil significantly increased according to four cropping systems (*A. histrix*/maize intercropping-yam rotation; *M. pruriens*/maize intercropping-yam rotation; 1-year fallow of *Andropogon gayanus-*yam rotation; maize-yam rotation) during the 2002 and 2004 cropping seasons in four villages in Benin.

Cropping system	Cropping season 2002	Cropping season 2004
	DM (t ha^−1^)	DM (t ha^−1^)
T0	4.1^c^	3.9^c^
TM	3.5^d^	3.2^d^
TMA	9.2^b^	8.3^b^
TMM	9.7^a^	8.8^a^

Means with the same letter within row are not significantly different (*P* > 0.05).

T0 (control 1): one-year fallow-yam rotation; TM (control 2): maize-yam rotation; TMA: *A. histrix*/maize intercropping-yam rotation; TMM: *M. pruriens*/maize intercropping-yam rotation; DM: dry matter.

**Table 6:** ANOVA, partial nested model of the effect of the four treatments on logarithmic transformed values of dry matter yields of  “Kokoro” yam (*Dioscorea rotundata*) (2002-2003 and 2004-2005, 4 sites, 32 farmers, Benin).

Source	DF	Adj. SS	Adj. MS	*F*	*P*
Site	3	0.4258	0.1419	*∗∗*	
Farmer (Site)	28	3.4833	0.1244	0.18	1.000
Replicate (Site)	96	42.3111	3.5259	27	0.000
Year	1	0.0002	0.0002	0.01	0.943
Treatment	3	224.0376	74.6792	5344.06	0.000
Site × Treatment	9	0.0291	0.0032	0.11	0.999
Treatment × Farmer (Site far)	84	2.2389	0.0267	1.62	0.001
Year × Farmer (Site)	28	6.933	0.2476	15.02	0.000
Year × Treatment	3	0.0114	0.0038	0.2	0.892
Year × Site	3	0.141	0.047	0.19	0.904
Year × Site × Treatment	9	0.1685	0.0187	1.14	0.334

Error	756	12.4598	0.0165		
Adjusted *R*-square (%)	94.24				

DF: degree of freedom; Adj. SS: adjusted sums of squares; Adj. MS: adjusted mean squares; *F*: Fisher's test; *P*: Fisher's probability test.

^*∗∗*^Denominator of *F*-test is zero.

**Table 7:** Dry matter (t ha^−1^) of yam tubers removed and yam shoots recycled in the 2002-2003 and 2004-2005 cropping seasons in four villages in Benin.

	2002-2003 cropping seasons					2004-2005 cropping seasons				
	T0	TM	TMA	TMM	LSD	T0	TM	TMA	TMM	LSD
*Yam DM removed (t ha* ^−*1*^)										
DM removed	5.09^b^	3.83^c^	7.20^a^	7.33^a^	0.51	4.34^b^	3.02^c^	8.00^a^	8.02^a^	0.55

*Yam shoots DM recycled (t ha* ^−*1*^)										
Yam shoots	1.27^b^	0.96^c^	1.80^a^	1.83^a^	0.13	1.09^b^	0.76^c^	2.00^a^	2.00^a^	0.14

Means with the same letter within row are not significantly different (*P* > 0.05).

DM: dry matter; LSD: least square difference at 5%.

T0 (control 1): one-year fallow-yam rotation; TM (control 2): maize-yam rotation; TMA: *A. histrix*/maize intercropping-yam rotation; TMM: *M. pruriens*/maize intercropping-yam rotation.

**Table 8:** Nitrogen, phosphorus, and potassium content (kg ha^−1^) dry matter of plant parts removed in the crop harvest and those returned to the soil in yam-based cropping systems (2002-2003 and 2004-2005 cropping seasons, four cropping system treatments, four village sites, 32 farmers, Benin).

		2002-2003 cropping seasons						2004-2005 cropping seasons					
		T0	TM	TMA	TMM	LSD	SD	T0	TM	TMA	TMM	LSD	SD
*Plant nutrients removed (kg ha* ^−*1*^)													
Yam tubers	N	19.35^b^	14.57^c^	27.37^a^	27.84^a^	1.95	2.98	16.49^b^	11.48^c^	30.41^a^	30.47^a^	2.08	3.18
	P	1.99^b^	1.49^c^	2.81^a^	2.86^a^	0.20	0.31	1.69^b^	1.18^c^	3.12^a^	3.13^a^	0.21	0.33
	K	21.39^b^	16.10^c^	30.25^a^	30.77^a^	2.16	3.30	18.23^b^	12.70^c^	33.61^a^	33.68^a^	2.30	3.52
Maize grains	N	0.00^b^	34.88^a^	34.43^a^	33.38^a^	2.27	3.47	0.00^c^	31.52^a^	27.68^b^	26.71^b^	2.03	3.11
	P	0.00^b^	5.30^a^	5.24^a^	5.08^a^	0.35	0.53	0.00^c^	4.79^a^	4.21^b^	4.06^b^	0.31	0.47
	K	0.00^b^	4.34^a^	4.28^a^	4.15^a^	0.28	0.43	0.00^c^	3.92^a^	3.44^b^	3.32^b^	0.25	0.39

*Plant nutrients recycled (kg ha* ^−*1*^)													
Yam shoots	N	14.01^b^	10.54^c^	19.81^a^	20.15^a^	1.41	2.16	11.72^b^	8.16^c^	21.60^a^	21.65^a^	1.48	2.26
	P	1.91^b^	1.44^c^	2.70^a^	2.75^a^	0.19	0.29	1.30^b^	0.91^c^	2.40^a^	2.41^a^	0.16	0.25
	K	17.57^b^	13.22^c^	24.85^a^	25.28^a^	1.77	2.71	14.65^b^	10.20^c^	27.01^a^	27.06^a^	1.85	2.83
Fallow stover	N	47.63^a^	0.00^b^	0.00^b^	0.00^b^	3.86	5.91	47.03^a^	0.00^b^	0.00^b^	0.00^b^	4.22	6.46
	P	5.26^a^	0.00^b^	0.00^b^	0.00^b^	1.23	1.89	5.09^a^	0.00^b^	0.00^b^	0.00^b^	1.27	1.94
	K	19.90^a^	0.00^b^	0.00^b^	0.00^b^	2.16	3.30	19.49^a^	0.00^b^	0.00^b^	0.00^b^	1.98	3.03
Maize stover	N	0.00^b^	31.87^a^	31.43^a^	30.47^a^	2.45	3.75	0.00^c^	33.45^a^	29.35^b^	28.28^b^	2.60	3.97
	P	0.00^b^	4.56^a^	4.51^a^	4.37^a^	0.39	0.60	0.00^c^	4.65^a^	4.08^b^	3.95^b^	0.45	0.68
	K	0.00^c^	17.48^ab^	18.57^a^	16.76^b^	1.86	2.84	0.00^c^	17.42^a^	15.30^b^	14.80^b^	1.42	2.17
Aeschy. stover	N	0.00^b^	0.00^b^	115.93^a^	0.00^b^	6.63	10.14	0.00^b^	0.00^b^	107.70^a^	0.00^b^	9.34	14.28
	P	0.00^b^	0.00^b^	8.15^a^	0.00^b^	0.97	1.49	0.00^b^	0.00^b^	8.76^a^	0.00^b^	0.69	1.05
	K	0.00^b^	0.00^b^	36.25^a^	0.00^b^	1.33	2.03	0.00^b^	0.00^b^	34.53^a^	0.00^b^	1.70	2.60
Mucuna stover	N	0.00^b^	0.00^b^	0.00^b^	138.92^a^	6.53	9.99	0.00^b^	0.00^b^	0.00^b^	133.25^a^	5.28	8.07
	P	0.00^b^	0.00^b^	0.00^b^	11.40^a^	1.81	2.77	0.00^b^	0.00^b^	0.00^b^	11.11^a^	1.61	2.46
	K	0.00^b^	0.00^b^	0.00^b^	39.73^a^	1.51	2.31	0.00^b^	0.00^b^	0.00^b^	39.68^a^	1.78	2.72

Means with the same letter within row are not significantly different (*P* > 0.05).

T0 (control 1): one-year fallow-yam rotation; TM (control 2): maize-yam rotation; TMA: *A. histrix*/maize intercropping-yam rotation; TMM: *M. pruriens*/maize intercropping-yam rotation; SD: standard deviation; LSD: least square difference at 5%.

**Table 9:** Total plant nitrogen, phosphorus, and potassium (kg ha^−1^) dry matter removed in the crop harvest and those returned to the soil in yam-based cropping systems (2002-2003 and 2004-2005 cropping seasons, four cropping system treatments, four village sites, 32 farmers, Benin).

		2002-2003 cropping seasons						2004-2005 cropping seasons					
		T0	TM	TMA	TMM	LSD	SD	T0	TM	TMA	TMM	LSD	SD
Total nutrients removal through harvest (kg ha^−1^)	N	19.35^c^	49.44^b^	61.80^a^	61.22^a^	2.91	4.46	16.49^c^	43.01^b^	58.09^a^	57.18^a^	2.90	4.44
	P	1.99^c^	6.80^b^	8.05^a^	7.93^a^	0.39	0.60	1.69^c^	5.97^b^	7.33^a^	7.19^a^	0.38	0.57
	K	21.39^b^	20.44^b^	34.53^a^	34.93^a^	2.16	3.30	18.23^b^	16.61^b^	37.05^a^	37.00^a^	2.31	3.54

Total nutrients recycled through plant biomass (kg ha^−1^)	N	61.64^c^	42.41^d^	167.17^b^	189.54^a^	10.68	16.33	58.75^c^	41.61^d^	158.66^b^	183.17^a^	11.59	17.72
	P	7.17^c^	6.00^c^	15.36^b^	18.52^a^	2.21	3.37	6.40^c^	5.56^c^	15.25^b^	17.47^a^	2.09	3.20
	K	37.47^b^	30.71^c^	79.66^a^	81.77^a^	3.24	4.95	34.14^c^	27.62^d^	76.84^b^	81.54^a^	3.94	6.03

Means with the same letter within row are not significantly different (*P* < 0.05).

T0 (control 1): one-year fallow-yam rotation; TM (control 2): maize-yam rotation; TMA: *A. histrix*/maize intercropping-yam rotation; TMM: *M. pruriens*/maize intercropping-yam rotation; SD: standard deviation; LSD: least square difference at 5%.

**(a):** 

	Akpéro		Gbanlin		Miniffi		Gomè	
Depth (cm)	0–10	10–20	0–10	10–20	0–10	10–20	0–10	10–20
	“Plinthosols”		“Plinthosols”		“Luvisols ferriques”		“Luvisols ferriques”	
Clay%	5.927	6.101	5.276	5.227	6.078	6.143	6.004	6.239
Silt%	10.482	10.755	5.425	5.446	6.329	6.568	15.950	16.089
Sand%	83.587	83.143	89.293	89.325	87.587	87.287	78.046	77.671
C%	0.996	0.909	0.686	0.672	0.756	0.723	0.625	0.587
N%	0.080	0.087	0.0575	0.059	0.061	0.061	0.0588	0.058
C/N	12.523	10.911	12.00	11.389	12.438	11.928	10.821	10.211
OM%	1.713	1.563	1.180	1.157	1.301	1.247	1.076	1.010
PH	6.364	6.095	6.020	6.278	5.934	6.020	5.934	5.848
Bray P	20.440	18.880	5.646	5.743	9.073	6.688	5.668	3.693
K	0.385	0.366	0.407	0.283	0.329	0.214	0.203	0.201

C%: soil carbon concentration; N%: soil nitrogen concentration; OM% (=1.72 × C%): soil organic matter content; C/N: index of biodegradability or ratio of soil carbon to nitrogen; Bray P (mg/kg-soil): soil phosphorus; K cmol kg^−1^: soil potassium.

**(b):** 

	Akpéro		Gbanlin		Miniffi		Gomè	
Depth (cm)	0–10	10–20	0–10	10–20	0–10	10–20	0–10	10–20
	“Plinthosols”		“Plinthosols”		“Luvisols ferriques”		“Luvisols ferriques”	
Clay%	5.363	5.666	5.020	5.006	5.913	5.811	5.780	5.959
Silt%	10.820	10.951	5.393	5.573	6.271	6.358	16.226	16.348
Sand%	83.816	83.381	89.581	89.423	87.815	87.834	77.997	77.697
C%	1.015	0.9165	0.669	0.655	0.754	0.684	0.617	0.557
N%	0.089	0.109	0.066	0.078	0.075	0.082	0.072	0.071
C/N	11.419	8.575	10.113	8.520	10.223	8.355	8.591	7.786
OM%	1.746	1.576	1.150	1.127	1.297	1.176	1.062	0.959
PH	6.993	6.733	6.650	6.897	6.555	6.650	6.555	6.441
Bray P	22.610	21.750	7.031	7.604	8.041	6.024	8.041	6.024
K	0.582	0.493	0.466	0.353	0.376	0.239	0.271	0.235

C%: soil carbon concentration; N%: soil nitrogen concentration; OM% (=1.72 × C%): soil organic matter content; C/N: index of biodegradability or ratio of soil carbon to nitrogen; Bray P (mg/kg-soil): soil phosphorus; K cmol kg^−1^: soil potassium.

**(c):** 

	Akpéro		Gbanlin		Miniffi		Gomè	
Depth (cm)	0–10	10–20	0–10	10–20	0–10	10–20	0–10	10–20
	“Plinthosols”		“Plinthosols”		“Luvisols ferriques”		“Luvisols ferriques”	
Clay%	6.509	6.752	5.455	5.999	6.245	5.882	5.567	5.390
Silt%	10.581	10.811	5.513	5.608	6.310	6.396	15.85	15.866
Sand%	82. 910	82.438	89.033	88.394	87.445	87.721	78.748	78.744
C%	1.1248	1.0583	0.732	0.685	0.781	0.771	0.635	0.608
N%	0.107	0.124	0.073	0.084	0.084	0.092	0.079	0.076
C/N	10.707	8.654	10.115	8.197	9.300	8.417	8.082	8.006
OM%	1.935	1.820	1.260	1.178	1.344	1.326	1.092	1.046
PH	7.371	7.221	7.112	7.237	7.034	7.087	6.997	7.031
Bray P	23.890	22.930	8.929	8.540	9.364	6.900	9.364	6.900
K	0.687	0.604	0.509	0.436	0.452	0.297	0.332	0.298

C%: soil carbon concentration; N%: soil nitrogen concentration; OM% (=1.72 × C%): soil organic matter content; C/N: index of biodegradability or ratio of soil carbon to nitrogen; Bray P (mg/kg-soil): soil phosphorus; K cmol kg^−1^: soil potassium.

**(d):** 

	Akpéro		Gbanlin		Miniffi		Gomè	
Depth (cm)	0–10	10–20	0–10	10–20	0–10	10–20	0–10	10–20
	“Plinthosols”		“Plinthosols”		“Luvisols ferriques”		“Luvisols ferriques”	
Clay%	6.180	6.539	5.724	6.045	6.371	6.191	5.561	5.440
Silt%	10.556	10.789	5.519	5.579	6.330	6.373	15.714	15.841
Sand%	83.264	82.673	88.758	88.376	87.299	87.436	78.725	78.719
C%	1.244	1.150	0.757	0.729	0.819	0.810	0.655	0.619
N%	0.127	0.138	0.083	0.086	0.088	0.094	0.085	0.078
C/N	9.959	8.425	9.224	8.545	9.239	8.457	7.707	7.944
OM%	2.140	1.978	1.303	1.253	1.409	1.393	1.126	1.064
PH	7.225	7.162	6.963	6.912	6.875	6.975	7.062	6.888
Bray P	23.110	22.700	10.015	10.393	11.665	7.755	11.665	7.755
K	0.746	0.663	0.552	0.494	0.479	0.338	0.367	0.315

C%: soil carbon concentration; N%: soil nitrogen concentration; OM% (=1.72 × C%): soil organic matter content; C/N: index of biodegradability or ratio of soil carbon to nitrogen; Bray P (mg/kg-soil): soil phosphorus; K cmol kg^−1^: soil potassium.

**(e):** 

Soil characteristics	Depth	T0	TM	TMA	TMM	LSD
Clay%	0–10 cm	5.821^c^	5.519^d^	5.944^b^	5.959^a^	0.111
	10–20 cm	5.928^c^	5.611^d^	6.006^b^	6.054^a^	0.124

Silt%	0–10 cm	9.546^a^	9.678^a^	9.522^a^	9.530^a^	ns
	10–20 cm	9.714^a^	9.807^a^	9.670^a^	9.645^a^	ns

Sand%	0–10 cm	84.628^a^	84.802^a^	84.534^a^	84.511^a^	ns
	10–20 cm	84.357^a^	84.584^a^	84.324^a^	84.301^a^	ns

C%	0–10 cm	0.766^b^	0.764^b^	0.818^b^	0.869^a^	0.037
	10–20 cm	0.723^b^	0.703^b^	0.780^a^	0.827^a^	0.033

N%	0–10 cm	0.064^d^	0.076^c^	0.086^b^	0.095^a^	0.003
	10–20 cm	0.066^c^	0.085^b^	0.094^a^	0.099^a^	0.004

C : N	0–10 cm	11.947^a^	10.087^b^	9.551^c^	9.032^c^	0.272
	10–20 cm	11.109^a^	8.309^b^	8.319^b^	8.343^b^	0.211

MO%	0–10 cm	1.317^b^	1.313^b^	1.408^a^	1.495^a^	0.063
	10–20 cm	1.244^c^	1.209^c^	1.342^b^	1.422^a^	0.057

Bray P (mg kg^−1^)	0–10 cm	10.210^c^	11.840^b^	13.430^a^	14.346^a^	1.126
	10–20 cm	8.750^c^	10.660^b^	11.410^ab^	12.290^a^	1.217

K^+^ cmol kg^−1^	0–10 cm	0.331^d^	0.424^c^	0.495^b^	0.536^a^	0.026
	10–20 cm	0.266^d^	0.330^c^	0.409^b^	0.453^a^	0.028

PH water	0–10 cm	6.063^c^	6.688^b^	7.129^a^	7.031^a^	0.055
	10–20 cm	6.060^c^	6.680^b^	7.144^a^	6.984^a^	0.053

Means with the same letter within row are not significantly different (*P* > 0.05).

C%: soil carbon concentration; N%: soil nitrogen concentration; OM% (=1.72 × C%): soil organic matter content; C : N: ratio of soil carbon to nitrogen; Bray P (mg/kg-soil): soil phosphorus; K^+^ cmol kg^−1^: soil potassium; LSD: least square difference at 5%; SD: standard deviation.

T0 (control 1): one-year fallow-yam rotation; TM (control 2): maize-yam rotation; TMA: *A. histrix*/maize intercropping-yam rotation; TMM: *M. pruriens*/maize intercropping-yam rotation; LSD: least square difference at 5%; ns: nonsignificant.

Data are the means.
